# Putting the Earth in Play: Environmental Awareness and Sports

**DOI:** 10.1289/ehp.114-a286

**Published:** 2006-05

**Authors:** Charles W. Schmidt

Since time immemorial, people have entertained themselves with sports. Sports are emblematic of health, with the best matches played by athletes in peak physical form. But ironically, even as sports promote health, they can also degrade the environment upon which good health depends. Whether played or watched, athletic endeavors have the potential to produce huge environmental “footprints” in terms of their use and abuse of natural resources. Ski slopes, for instance, disrupt fragile alpine ecosystems, while snowmobiles spew exhaust fumes into the air. Golf courses sprawl across the land, and consume large amounts of pesticides and water, while parking lots for stadiums and arenas produce vast paved surfaces. And major sports events use energy, emit greenhouse gases, and produce voluminous trash. The 2006 Super Bowl in Detroit produced 500 tons of the greenhouse gas carbon dioxide (from transportation and utility usage), while the 2004 Summer Olympics in Athens produced half a million tons in two weeks—roughly comparable to what a city of 1 million people would emit over a similar period. Each match during the 2006 World Cup this summer will use up to 3 million kilowatt-hours of energy (similar to the annual consumption of 700 European households), and produce an estimated 5–10 tons of trash.

These impacts have spawned an environmental movement with two broad goals: to reduce the ecological footprint of sports activities, and to exploit the popularity of sports to raise environmental awareness in general. “Like any other sector, sport has environmental consequences,” says David Chernushenko, president of Green and Gold, a sports sustainability consulting firm in Ottawa, Canada, and author of the first book on the subject—*Greening Our Games*, published in 1994. “But sports are also heavily impacted by degraded environments, and that’s important to an athlete who can’t run on smog days, or to those in the golf industry who get told they can’t build a new course because bad practices have tarred their image. So, sports create opportunities to produce leaders for better environmental practice.”

## UNEP at the Fore

The sports sustainability movement now encompasses numerous environmental groups, businesses, and nongovernmental organizations (NGOs). The UN Environment Programme (UNEP), a veteran influential player in this arena, was among the first to get involved. In 1994, UNEP created a Sports and Environment Program, and charged it with promoting environmental awareness through sports as well as the design of sustainable sports facilities and equipment.

Currently headed by Eric Falt, UNEP’s director of communications and public information in Nairobi, Kenya, the program has fostered numerous initiatives. In 1994, the Centennial Olympic Congress of Paris established the environment as a “third pillar” of the Olympic charter, along with sport and culture. In a pivotal milestone, UNEP teamed with the International Olympic Committee (IOC) in 1995 to host the first World Conference on Sport and Environment, held in Lausanne, Switzerland. Participants there created a Sport and Environment commission within the IOC. The latest world conference, held in Nairobi in November 2005, yielded the Nairobi Declaration on Sport, Peace, and Environment, which calls upon the IOC and national Olympic committees to act as leaders in promoting environmental sustainability through sports.

UNEP has also organized three meetings of the Global Forum for Sport and Environment (G-ForSE) since 2001, in which sports stakeholders in and beyond the Olympic Movement review their contributions to sustainable development. At the July 2005 Sports Summit for the Environment, a G-ForSE meeting held in Aichi, Japan, participants signed the Joint Declaration on Sports and the Environment, in which they pledged to help address environmental problems and create a sustainable world society through sports.

UNEP has also worked with the IOC to develop an “Agenda 21” for the Olympic Movement based on environmental sustainability guidelines created by delegates at the 1992 UN Conference on Environment and Development. By adopting its own Agenda 21, the IOC committed itself to encouraging sustainability among its member nations and sports governing bodies. This agenda is being used by several National Olympic Committees for sustainable development work at the national level.

NGOs working in this area include the Global Sports Alliance (GSA), based in Tokyo. The GSA, which is supported by UNEP, partners with numerous sports groups including the IOC to help create an environmentally aware sports culture. GSA members try to spread environmental awareness in part by sending “ecoflags” to schools and sports clubs, which these organizations fly during games to affirm ecological commitments. The GSA also sponsors several projects and, with UNEP, the G-ForSE. [For more information on the GSA, see “EHPnet: Global Sports Alliance,” p. A279 this issue.]

## Greening of the Olympics

The 1994 Winter Olympics in Lillehammer, Norway, are now viewed as the first attempt to create a “green” Olympic Games. Local activists in Lillehammer successfully forced the country’s Olympic Organizing Committee (OOC) to make changes based on environmental concerns. Because of their actions, a speed skating rink was redesigned to avoid impacts to a nearby bird sanctuary, and officials agreed to an environmental plan emphasizing renewable building materials and energy-efficient heating and lighting for facilities, trash recycling, and arena designs that harmonize with the local landscape.

Since Lillehammer, the IOC has tried to make the Olympics a showcase for environmental sustainability. With the 1999 adoption of the Olympic Movement’s Agenda 21, any country that wants to host the Olympics has to produce a strategic environmental assessment to accompany its bid. David Crawford, a Winnipeg, Canada–based sustainability advisor to OOCs, says these assessments must describe environmental commitments around energy use, water consumption, waste generation, and sustainable building construction, in addition to social commitments to include local communities in the planning process. “If you look at who won the last three Olympic bids—Beijing in 2008, Vancouver in 2010, and London in 2012—you see environmental assessments played a major strategic role in that success,” he says.

Intent and implementation aren’t one and the same, however. Despite successful bids, some host cities have found their Olympic sustainability obligations hard to meet. The Athens Games, for instance, are widely viewed as an environmental failure, particularly with respect to sustainable construction and green energy. Despite Athens’ commitment to use 100% renewable energy during the Games, almost all the energy expended there ultimately came from non-renewable sources.

Beijing could also have trouble meeting its environmental obligations. The city’s air quality ranks among the world’s worst—indeed, the highest nitrogen dioxide levels in any city are found there. Exposure to Beijing’s air can therefore irritate and damage the respiratory tract, posing an obvious hazard to competing athletes. To prepare its Olympic bid, Beijing promised to achieve 230 “blue sky” days per year, meaning days when air quality is “good or moderate.” To achieve this, the city ordered the Shougang Corporation, a major steel maker, to move its coal-fired smelters—and some 120,000 employees—to a small island in neighboring Hebel province. City officials also imposed tighter auto emissions standards two years ahead of national implementation. These measures have produced some success: Beijing’s air quality has improved, and the city claims it achieved 234 blue sky days in 2005. But air quality in January 2006 was the worst in six years, with only nine blue sky days reported.

The IOC’s choice of Beijing underscores the notion that environmental sustainability—while important—isn’t a deal breaker for host city selection. “Let’s not kid ourselves,” Crawford says. “The Olympic Movement is global, the Games can’t always be held in the same continents. Beijing’s air quality is bad, so the Chinese are using the Olympics for a public environmental education campaign. They are keenly aware they have a problem; the Olympics can be a positive catalyst for change.”

As for the Torino Winter Olympics, a full picture of its environmental performance is now emerging. Falt acknowledges some problems at Torino: for instance, bobsledding created environmental and sustainability challenges, he says. The bobsled track, which Falt describes as a “huge fridge in the mountains,” has a coolant system containing 48 tons of ammonia that could harm wildlife and human health if leaked. What’s more, the track’s annual maintenance cost of up to US$1.1 million will likely exceed visitor-generated revenue. On a more positive note, in a press release dated 1 March 2006, UNEP executive director Klaus Töpfer commended Torino for building skating rinks and other facilities in the city center to promote continued use. He also lauded efforts to limit erosion and runoff from ski slopes, and the use of renewable materials and energy-efficient systems in building construction.

## The Carbon Counting Game

Two of the environmental programs employed by Torino’s OOC are particularly notable. One is its use of the European Union’s Eco-Management and Audit System, through which registered organizations in Europe evaluate, report on, and improve their environmental performance. Twenty-nine Olympic sites in Torino, including training facilities and buildings in the Olympic village, were built by companies registered with the system. The other notable program is Heritage Climate Torino, which strives to offset the estimated 300,000 tons of greenhouse gases released during the two-week event. According to Ugo Pretato, the Torino OOC head of environmental programs, the Regional Public Administration in Piedmont (the Italian province of which Torino is the capital) allocated approximately US$6 million for carbon credits linked to several greenhouse gas mitigation projects, including a reforestation project in Mexico, renewable energy projects in India and Sri Lanka, and an energy efficiency scheme in Eritrea. “The expectation is that Heritage Climate Torino will become more developed over time,” says Pretato. “We hope our example will be followed by other big sports events in the future.”

Offsetting carbon emissions from spectator events is a noble gesture, but also one that’s new and untested. An obvious question concerns the amounts of greenhouse gases that events like the Olympics actually produce. Quantifying them is no easy task, says Mark Bain, director of Cornell University’s Center for the Environment. “Do you count the extra flights, hotel stays, and changes in personal habits?” he asks. “It’s not just the spatial boundaries you have to consider, it’s also the downstream and upstream consequences to the carbon cycle. I think lots of organizations want to say they’re making up for their environmental effects, but most haven’t fully considered what this actually means.”

For his part, Pretato says the Torino OOC counts all transportation to and from the Olympics, including air travel, in addition to energy consumption by all Torino venues and stadiums. Data collection is still ongoing, he says.

The U.S. National Football League (NFL) also plays the carbon counting game. Seeking to offset the greenhouse gas emissions of Super Bowl XL, played 5 February 2006 in Detroit, the NFL consulted with scientists at Oak Ridge National Laboratories and Princeton University, who concluded that an acre planted with 250 native Michigan trees would absorb 75 tons of carbon over the trees’ life span. The NFL ultimately planted 2,500 trees over 10 acres in Michigan to offset the Super Bowl’s carbon emissions, a number that Jack Groh, director of the NFL Environment Program, says far exceeded what was necessary to mitigate the game’s climate impact.

Meanwhile, organizers with the 2006 World Cup, which overtakes Frankfurt, Germany, in June, are striving for “climate neutrality” (i.e., zero impact), which they hope to achieve by offsetting the expected 100,000 tons of greenhouse gas emissions with investments in renewable energy and energy-efficient technology. Climate neutrality is just one aspect of the World Cup’s extensive environmental agenda, however. As described in *Green Goal: Environmental Goals for the FIFA 2006 World Cup*, published by the Institute for Applied Ecology in Berlin, additional objectives are found in the areas of water use, recycling, energy efficiency, and traffic mitigation. World Cup organizers and The Coca-Cola Company have agreed to use recyclable cups at the event. And rain will be channeled into storage systems designed to provide water for cleaning playing surfaces and parking lots, in addition to toiletry needs. Indeed, organizers plan to save as much as 10,000 cubic meters of drinking water by installing the latest in water-free urinals.

Major sports events like the Olympics, the Super Bowl, and the World Cup generate large environmental footprints over short durations. But what of the day-to-day sports played by billions of ordinary people? Many are environmentally benign. But others do have potentially serious environmental consequences. Here are some examples.

## Skiing: A Slippery Slope

Skiing—a sport whose very existence is in some places threatened by global warming—can produce substantial environmental impacts. Ski slopes disrupt the natural landscape, sometimes harmfully so, according to Ryan Bidwell, executive director of Colorado Wild, a Durango-based environmental group. “Downhill ski terrain typically gets carved into ecologically sensitive high-alpine environments,” he explains. “And these areas have short growing seasons, so they aren’t quick to recover.” Trail building contributes to erosion because it removes trees and shrubs that anchor soils. Other negative impacts come from snow making, which could become more prevalent in some areas because of global warming. Snow making diverts natural waters, altering the normal flows of rivers and streams that supply the necessary water, and resulting in dry stream beds, effects on irrigation, and consequences for species that depend on stream flow.

Some streams in Colorado and other western states are contaminated with acids and metals such as cadmium, copper, lead, and zinc—a legacy of the region’s mining industry. Snow made from these sources might contaminate otherwise pristine areas, Bidwell says. In one high-profile case, owners of the Arizona Snowbowl Ski Resort will soon make snow from treated wastewater. Their announcement of doing so drew a sustained outcry from the local Navajo population, which views the surrounding San Francisco Peaks as a sacred natural shrine. But these objections were overruled by U.S. District Court judge Paul Rosenblatt in January 2006, clearing the way for waste-water snow making to begin. Snowbowl officials say the wastewater poses no health risks, but caution skiers against eating the snow, which—according to the resort’s website—contains residues from “animals, litter, boots, saliva, petroleum products, etc.”

Another key issue concerns the ongoing expansion of western ski resorts on public lands. In these cases, resorts expand until they buttress private land boundaries, attracting the development of multimillion-dollar homes built by those who can pay for residential slopeside access. Construction of these homes in delicate high-alpine areas brings numerous problems, however, including erosion, air emissions, impacts to endangered species, and water withdrawals.

To improve their environmental performance, 178 U.S. resorts have endorsed the National Ski Areas Association’s Sustainable Slopes Initiative, a collection of environmental best practices for ski owners and operators that was adopted in June 2000. The initiative promotes 21 principles in areas such as planning design, water and energy use, recycling, air quality, and forest management. A total of 71 resorts also participate in “Keep Winter Cool,” an initiative sponsored by the National Ski Areas Association and the Natural Resources Defense Council that promotes energy efficiency in ski operations and also supports anti–climate change legislation.

While notable, these initiatives have critics who counter that they don’t go far enough. Bidwell, for instance, blasts the Sustainable Slopes Initiative, suggesting it does little to address secondary impacts from land development and the destructive consequences of snow making, which he says pose the greatest environmental damage from skiing. “The charter has no accountability and no system to document whether resorts follow through on any of their proposals,” he adds.

To counter these perceived gaps, the Ski Area Citizens’ Coalition, also based in Durango, produces an annual “Ski Areas Environmental Scorecard,” which grades 77 resorts on their performance in areas such as energy efficiency, reduced habitat impacts, and efforts to expand operations within existing area boundaries. In the 2005/2006 scorecard, the coalition reported that only 50% of resorts supported legislation to combat climate change. Just 21% used alternative fuels such as biodiesel, 31% used wind or solar power, and 60% supported mass transit programs.

## Teed Off at Golf

Many golfers prefer their courses to be blanketed in velvety green grass, regardless of where the course is sited, be it the beach, the desert, or a naturally lush locale. Golf courses thus must be intensively coddled with lots of water and lots of pesticides. Each of the more than 17,000 golf courses in the United States alone can consume hundreds of thousands of gallons of water per day. And according to Stuart Cohen, president of the Wheaton, Maryland–based consultancy Environmental & Turf Services, golfing greens are among the most intensive nonagricultural users of pesticides.

Cohen says approximately 50 pesticide active ingredients are commonly used by the golf industry, although the number typically used on any one course is much lower, ranging from 4 to 12 per year, depending on location. Among the chemicals used are chlorpyrifos, an organophosphate insecticide whose residential uses are banned by the EPA due to developmental hazards; carbaryl, a carbamate insecticide; and chlorothalonil, an organochlorine fungicide.

Despite high-level use, documented cases of environmental harm from pesticides on golf courses are rare. In one instance, dating back to the mid-1980s, hundreds of Canadian geese were found dead on the Seaway Harbor fairways in Hempstead, New York—apparently poisoned by diazinon, an organophosphate insecticide that was subsequently banned from golf course applications in 1990 and from all residential uses in 2005. Another organophosphate pesticide—fenamiphos—has produced fish kills when washed into waterways from golf courses after heavy rains. Fenamiphos is now being phased out in Florida, where these fish kills have occurred, and a nationwide ban will be complete in 2007, Cohen says.

Cohen has conducted the largest survey to date of water quality impacts from U.S. golf courses, which was published in the May–June 1999 issue of the *Journal of Environmental Quality*. This review of 17 studies performed on 36 golf courses found little evidence of environmental harm, however. Cohen wrote, “None of the authors of the individual studies concluded that toxicologically significant impacts were observed,” but he also concluded that “there are major gaps in this review, particularly in the mid-continent area.” He is now updating and expanding this survey with funding from the U.S. Golf Association and the Golf Course Superintendent Association of America.

Cohen believes that when properly applied, golf course pesticides pose a low risk of exposure to players and nearby residential populations. This is in part, he says, because turf is a dense “living filter” with a thatch underlining that not only grips pesticides but also prevents them from leaching into groundwater. The turf system is also microbially active, and thus tends to degrade pesticides.

J. Marshall Clark, a professor of entomology at the University of Massachusetts Amherst, agrees. He and PhD student Ray Putnam have performed extensive risk assessments as part of Putnam’s thesis showing that dermal exposure—particularly through the lower legs, thighs, and lower arms—is the main way that players are exposed to golf course pesticides. Clark says his additional dosimetry studies, which measured excreted pesticides and metabolites in urine, have shown that the doses absorbed by players are far beneath any hazardous level. “People used to think hand-to-mouth was the main exposure route—for instance, golfers putting golf tees in their mouths,” he says. “But studies have dispelled that notion; the amount of hand-to-mouth activity on golf courses is small. Also, we find that hands are often well protected, and players are always wiping their hands off when they play, which removes the residues.”

Some environmentalists aren’t convinced, however. Jay Feldman, executive director of Beyond Pesticides, a Washington, DC–based environmental group, believes the exposure scenarios considered by the EPA thus far are incomplete, particularly as they apply to young golfers and chlorpyrifos. “The EPA’s view is that children don’t play golf, so golf courses can continue using chlorpyrifos,” he says. “But if you look at the U.S. Golf Association’s own statistics, you see kids are playing golf more and more. We think childhood risks should be taken into account by the EPA for all turf chemicals and for chlorpyrifos in particular.”

Water conservation is perhaps a more pressing problem for golf courses, and many facilities are trying to conserve. According to the 2001 report *Water Right: Conserving Our Water, Preserving Our Environment*, published by the International Turf Producers Foundation, the U.S. Golf Association has spent more than $18 million since 1982 seeking solutions to environmental issues related to golf, including the development of new grasses that require less water and pesticides, improved irrigation techniques, and use of alternative water sources, such as treated wastewater and storm runoff collected in storage ponds.

## NASCAR: The New Baseball

NASCAR racing is the fastest growing sport in America. In 2004, a total of 3.5 million fans watched races sponsored by NASCAR (the National Association of Stock Car Racing). Once concentrated mainly in the Deep South, NASCAR now lays claim to audiences throughout the United States, and even in Mexico. While a day at the races might seem like good clean fun, NASCAR can also produce significant environmental problems, including noise pollution, polluted runoff from tracks and parking lots, and reliance on an old health villain: leaded gas.

Although the EPA phased leaded gas out of the consumer market more than 30 years ago, its use in stock cars has gone on with the agency’s blessing—an exemption was written into the Clean Air Act. Lead lubricates engines, helping them run smoothly, but it’s also a neurotoxicant that can lower IQ, particularly among young children. In December 2005, a draft EPA document titled *Air Quality Criteria for Lead* stated that leaded fuel may pose a serious risk to residents living in the vicinity of racetracks, fuel attendants, racing crew and staff, and spectators.

In a pilot study published in the February 2006 issue of the *Journal of Occupational and Environmental Hygiene*, Joseph O’Neil of the Indiana University School of Medicine and colleagues found elevated blood lead levels among some mechanics and crew members of a NASCAR race team. Specifically, the median blood lead level in 47 tested individuals was 9.4 micrograms per deciliter, which approaches the EPA’s own risk threshold of 10 micrograms per deciliter, over which toxic effects can be expected. Nineteen of those individuals had blood levels at the EPA threshold.

For years, the EPA has urged NASCAR to quit leaded gas voluntarily. The industry claimed it was trying to find replacements, but also insisted the ones that were available lowered performance and harmed engines. But in January 2006, under pressure from Clean Air Watch, a Washington, DC–based environmental group, NASCAR finally relented. The industry will begin using a lead-free fuel made by Sunoco called 260 GTX by 2008.

## Other Impacts

Golfing, skiing, and stock car racing are not the only sports that present problems for the environment, however. Fishing, considered a competitive sport by some and a recreation by others, is being shown to have significant impacts on fish populations. A study in the 27 August 2004 issue of *Science* showed that recreational catches represented almost a quarter of catches of fish species identified by the U.S. government as species of concern for declining populations. Other water sports also have significant environmental impacts. Conventional outboard motors and personal water craft may release as much as 30% of their fuel into the water unburned. Recreational marine engines contribute a high percentage of hydrocarbon emissions to the air. And boating activities can have dire effects on estuaries that serve as nurseries for many fish species. [For more information on these impacts, see “The Environmental Pain of Pleasure Boating,” *EHP* 111:A216–A223 (2003).]

One group is trying to bring awareness to these issues. On 3 April 2006, the *Earthrace*, an 80-foot trimaran billed as the “world’s coolest boat,” was launched in Auckland’s Waitemata Harbour. The *Earthrace* project is a bid to break the world record for circumnavigating the globe (24,000 nautical miles) in a powerboat, using only renewable fuel. The project includes an 18-month tour calling at 60 major cities, promoting biodiesel and raising awareness about sustainable use of resources along the way. Sponsored by more than 200 marine supply companies, the boat is a showcase of environmentally friendly technologies such as low-emission engines, nontoxic antifouling paint, and efficient hull design. *Earthrace* skipper Paul Bethune said in a February 2006 press release, “By racing an awesome-looking boat on this fuel around the world, we hope to raise public awareness of the need to take alternative fuels seriously, as well as [display] incredible advances in the ways marine technology now coexists harmonically with marine ecology.”

The environmental footprint of sports extends beyond the activities themselves. The manufacture of sports clothing and equipment also exerts potential environmental impacts, mainly worker exposure to production chemicals and plant releases of dyes and wastewater, says André Gorgemans, secretary general of the World Federation of the Sporting Goods Industry (WFSGI) in Verbier, Switzerland. Of particular concern, Gorgemans says, are uses of polyvinyl chloride (PVC)—a type of plastic linked equivocally to testicular cancer and more definitively to many other health effects—for making soccer and cricket balls, footwear, bats, helmets, gloves, shin pads, and other sports items. Many countries around the world have been phasing out PVC (which also has numerous other uses in construction and plumbing) since toxicity issues first arose in the 1980s.

Today, the WFSGI discourages the use of PVC and hundreds of other toxic chemicals—including metals, dyes, and ozone-depleting chemicals—by sports manufacturers. All these chemicals are listed in the organization’s 2003 policy document titled *Guidance on Restricted Substances in Sports Footwear, Apparel, and Accessories*. Restricted substances, as described by the WFSGI, include chemicals that have been either legally banned by national governments in the European Union and elsewhere, or subjected to voluntary restrictions by nongovernmental ecolabeling schemes.

Frank Henke, global director of social and environmental affairs at adidas-Salomon and vice chairman of the WFSGI Committee for Corporate Social Responsibility, which produces the restricted substances list, says most “branded companies,” such as Nike and adidas, adhere to it. But he acknowledges that PVC and other restricted substances are still used by smaller manufacturers in developing countries. Henke declined to identify these manufacturers, however.

In addition to issues of the components of sports equipment, the manufacture of such equipment also plays into issues of obsolescence and waste. As any parent with a cluttered garage knows, used sports equipment can pile up quickly. Multiply one garage by all the others out there, and it’s easy to get a picture of how much waste sports activities can produce. Although equipment is occasionally passed down to siblings or resold, seldom is it recycled. Two projects of the GSA are working to remedy this situation. Sports-eco.net is a grassroots initiative to reduce, reuse, and recycle sports equipment, particularly the 30 million tennis balls that are manufactured every year. The program collects the balls and distributes them to schools for use on chair and table legs to muffle noise. The GSA website states, “By sending used tennis balls to primary and junior high schools around the country, we are reducing noise levels and creating a better atmosphere to learn, we are helping hearing impaired children (hearing aids are sensitive to sudden loud noises), and we are teaching a valuable environmental lesson.”

Similarly, the Igfy Corporation in Japan has pioneered a program to carry out the GSA mission. Called RECYCL’art, the program offers information and workshops on how to turn used sports equipment—including tennis rackets, balls, and shoes—into art. The program supplies special boxes that can be set up at schools, stores, and sporting events for collecting old or unused sports equipment for recycling.

Some sports manufacturers themselves seem to be catching on to the idea. Nike offers a program called Reuse-A-Shoe in which used athletic shoes are collected, deconstructed, and turned into “Nike Grind,” actually three different materials, each used in a different way to resurface soccer and football fields, basketball and tennis courts, tracks, and playgrounds.

## A Sporting Chance

In many ways, the emerging environmentalism in sports is highly collaborative, says Falt. “We don't think it’s useful to blame specific sports or federations for environmental problems,” he says. “Confrontation doesn’t work. We need to engage these entities directly.”

Meanwhile, the sports and environment movement continues to grow. Falt points out that during the early 1990s, the linkage between them had barely been made. But now, sports and the environment are indelibly linked—from the glitziest athletic spectacles, played out on the world stage, to the everyday games played by billions of ordinary people—and from this current generation of sports enthusiasts, a new generation of environmentalists may be emerging.

## Figures and Tables

**Figure:**
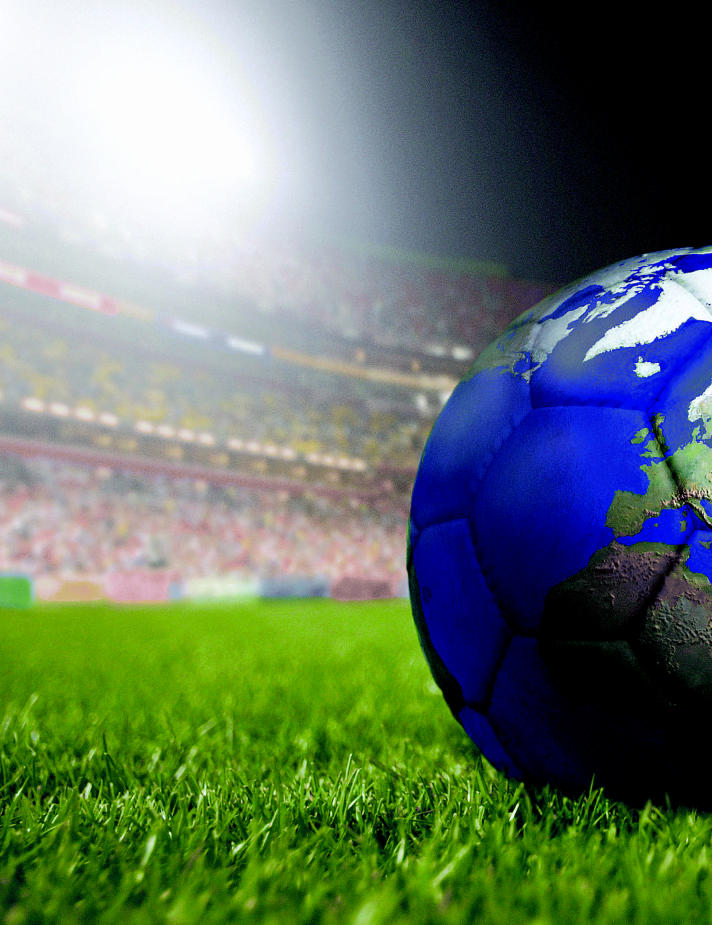


**Figure:**
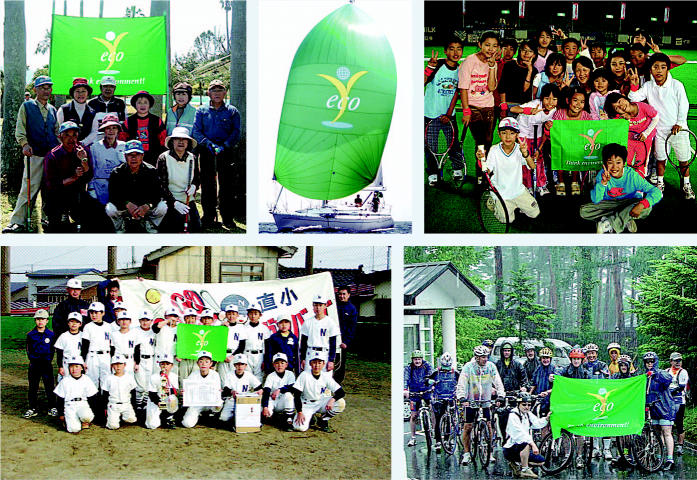
Doing the wave. The ecoflag, a symbol of environmental awareness in sports, flies at sports events.

**Figure:**
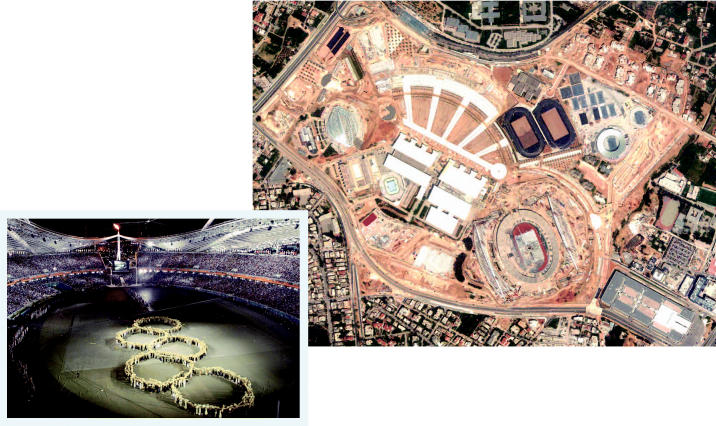
Failure to medal. From initial construction of facilities such as the Olympic Sports Complex (above) through the closing ceremony (left), the 2004 Athens Summer Olympics are widely viewed as an environmental failure, plagued by problems such as poorly designed venues and inefficient energy use.

**Figure:**
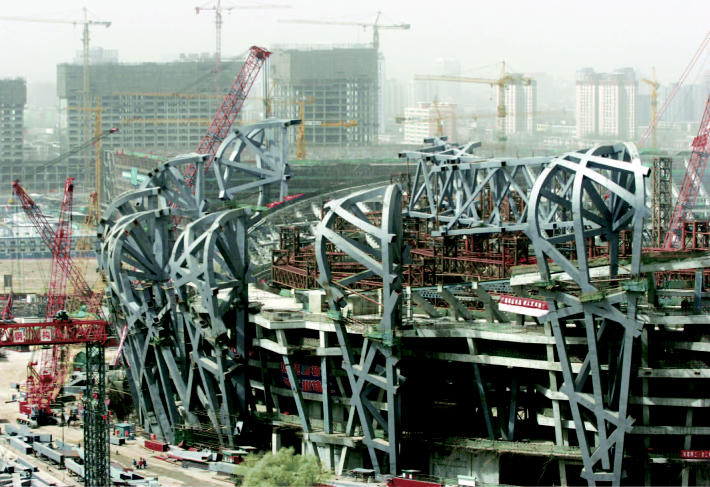
Competitive environment. Cranes add segments to the National Olympic Stadium, dubbed the “Bird Cage,” being built in Beijing for the 2008 Olympics. China’s bid to host the games included a strategic environmental assessment describing commitments such as sustainable construction.

**Figure:**
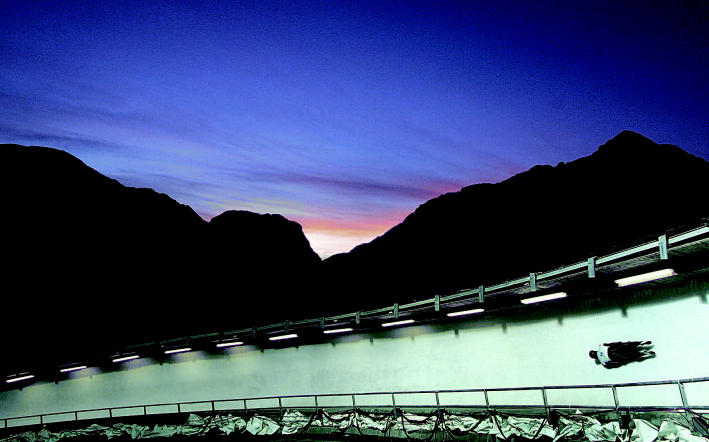
On thin ice? The bobsledding track used at the 2006 Winter Olympics in Torino contains 48 tons of ammonia that could harm wildlife if leaked.

**Figure:**
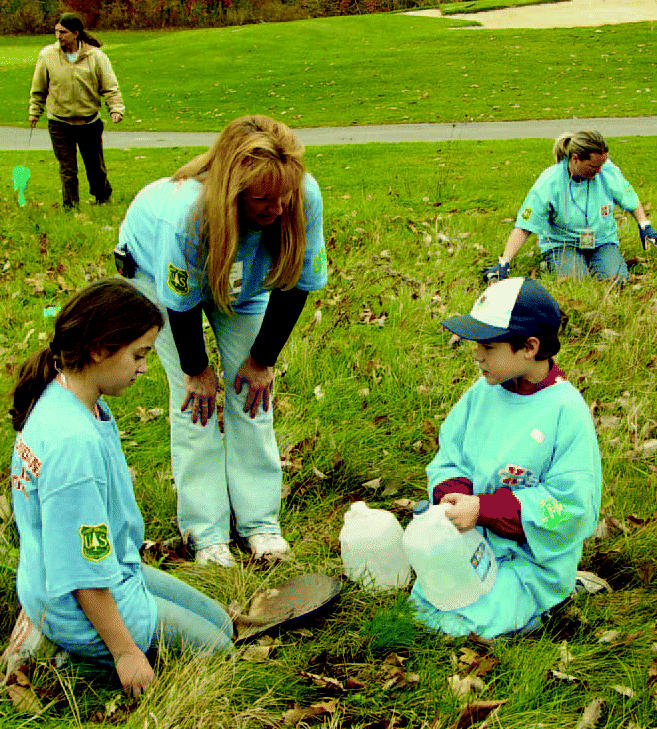
Paying to play. Children plant trees in the Detroit area as part of a carbon mitigation project for Super Bowl XL.

**Figure:**
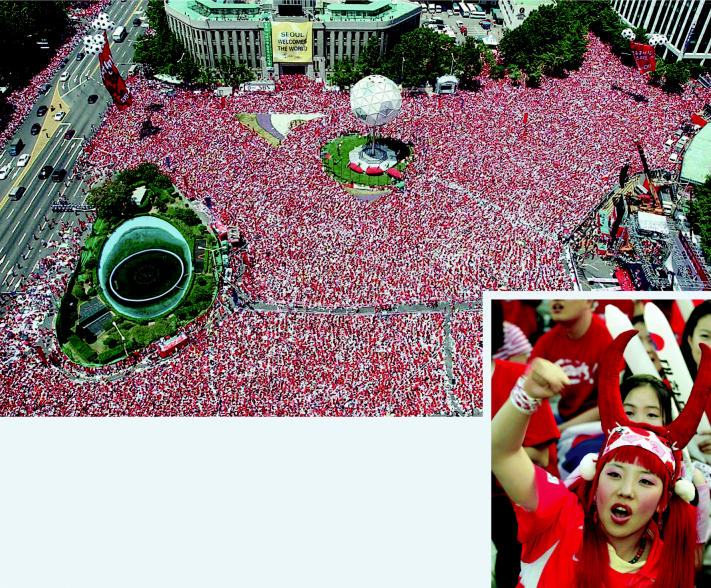
. . . And the crowd goes wild. South Korean soccer fans gathered in Seoul to watch a live broadcast of the 2002 World Cup quarter final match. The 2006 World Cup is striving for zero impact on the environment through greenhouse gas emission offsets, recycling, and traffic mitigation.

**Figure:**
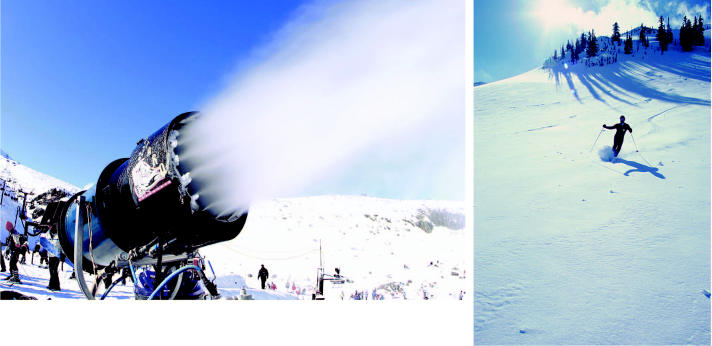
Snowball effect. With greater attention focused on the impacts of skiing, perhaps more resorts will sign on to—and honor—eco-friendly programs such as the Sustainable Slopes Initiative.

**Figure:**
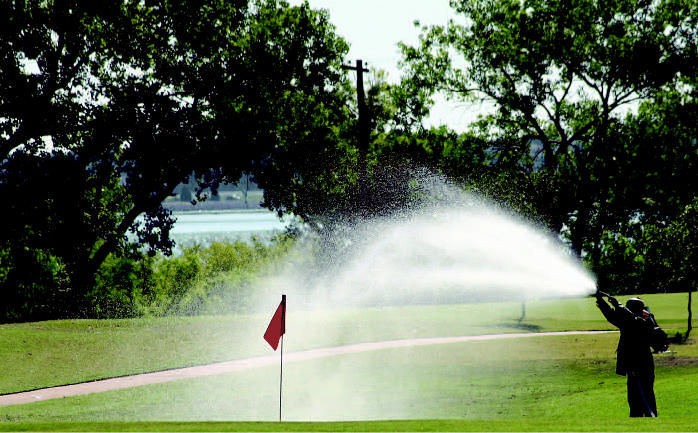
Missing the green. Golf courses are huge consumers of water and pesticides, raising environmental concerns for both those who play and those who live near them.

**Figure:**
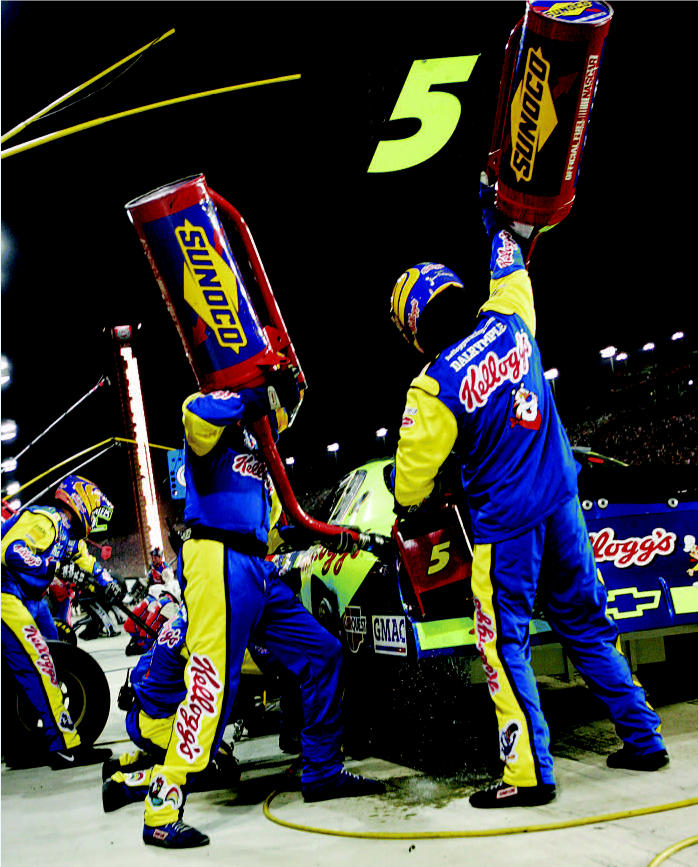
Fast track to cleaner air. Under pressure from environmental groups to phase out leaded gas, NASCAR will require stock cars to use a lead-free fuel made by Sunoco beginning in 2008.

**Figure:**
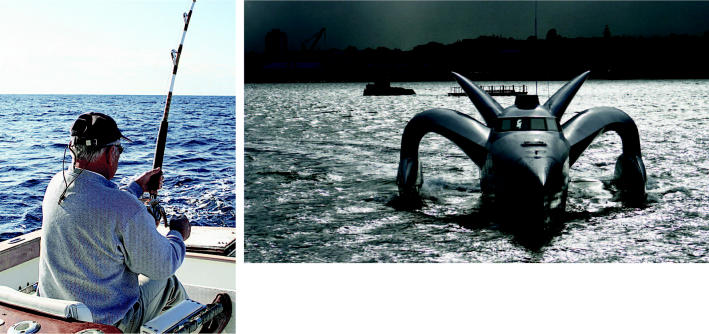
Reeling in a big one for the Earth. Sport fishing and boating have had many negative ecological impacts, but the *Earthrace* project (above), which is attempting to break the record for circumnavigating the globe in a boat run on renewable fuels, aims to show that marine sports can be less damaging to the environment.

**Figure:**
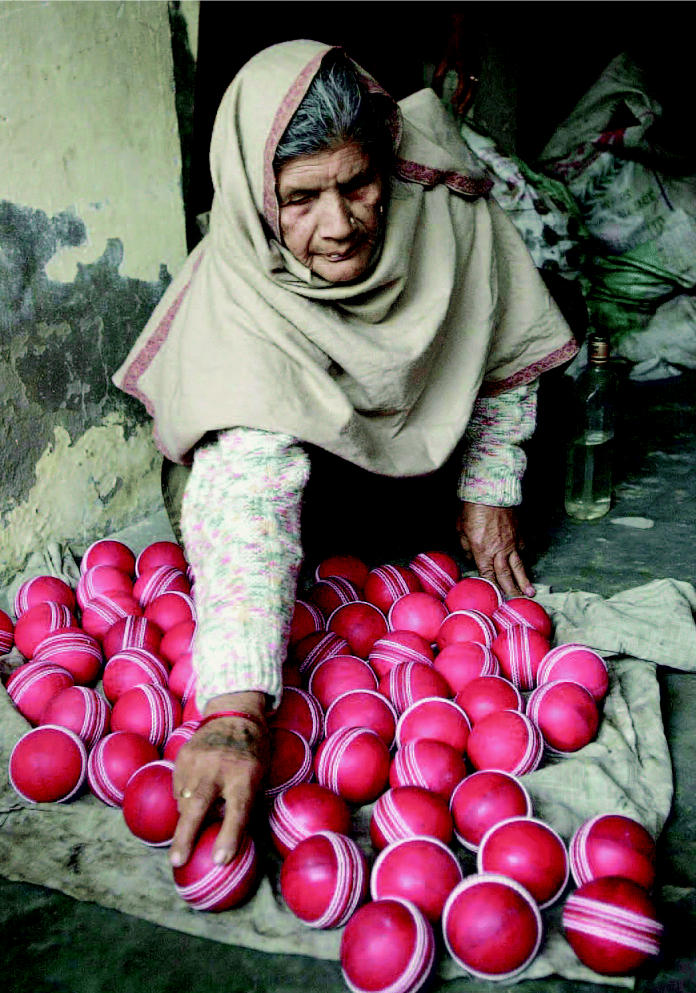
A sticky wicket. Although many large companies voluntarily restrict or ban the use of toxic chemicals in their sporting equipment, smaller manufacturers in developing countries still use chemicals, such as the PVC used in cricket balls, that may harm human health.

